# PBX1 Attenuates Hair Follicle-Derived Mesenchymal Stem Cell Senescence and Apoptosis by Alleviating Reactive Oxygen Species-Mediated DNA Damage Instead of Enhancing DNA Damage Repair

**DOI:** 10.3389/fcell.2021.739868

**Published:** 2021-11-15

**Authors:** Yuan Wang, Yutong Sui, Aobo Lian, Xing Han, Feilin Liu, Kuiyang Zuo, Mingsheng Liu, Wei Sun, Ziyu Wang, Zinan Liu, Fei Zou, Rifeng Lu, Minghua Jin, Haiying Du, Kan Xu, Xiaomei Liu, Jinyu Liu

**Affiliations:** ^1^ Department of Toxicology, School of Public Health, Jilin University, Changchun, China; ^2^ Eye Center, The Second Hospital of Jilin University, Changchun, China; ^3^ Department of Neuroscience, Mayo Clinic, Jacksonville, FL, United States; ^4^ Department of Neurovascular Surgery, First Hospital of Jilin University, Changchun, China

**Keywords:** PBX1, hair follicle (HF), mesenchymal stem cells, senescence, apoptosis, DNA damage and repair

## Abstract

Tissues and organs undergo structural deterioration and functional decline during aging. DNA damage is considered a major cause of stem cell senescence. Although stem cells develop sophisticated DNA repair systems, when the intrinsic and extrinsic insults exceed the DNA repair capacity, cellular senescence, and age-related diseases inevitably occur. Therefore, the prevention and alleviation of DNA damage is an alternative to DNA repair in attenuating stem cell senescence and preventing age-related diseases. Pre-B-cell leukaemia homeobox 1 (PBX1) participates in maintaining the pluripotency of human embryonic and haematopoietic stem cells. Our recent studies showed that PBX1 promotes hair follicle-derived mesenchymal stem cell (HF-MSC) proliferation, decreases cellular senescence and apoptosis, and enhances induced pluripotent stem cell generation. Whether PBX1 attenuates HF-MSC senescence and apoptosis by alleviating DNA damage or by enhancing DNA repair remains unknown. In this study, we aimed to determine the effects of PBX1 on the intrinsic ROS or extrinsic H_2_O_2_-induced cellular senescence of HF-MSCs. To this end, we generated HF-MSCs overexpressing either PBX1, or poly (ADP-ribose) polymerase 1, or both. Our results showed that PBX1 overexpression attenuates HF-MSC senescence and apoptosis by alleviating reactive oxygen species (ROS)-mediated DNA damage instead of enhancing DNA repair. This is the first study to report that PBX1 attenuates stem cell senescence and apoptosis by alleviating DNA damage. It provides new insight into the mechanism of stem cell senescence and lays the foundation for the development of strategies for age-related disease prevention and treatment, and in particular, hair follicle repair and regeneration.

## Introduction

Tissues and organs undergo structural deterioration and functional decline during aging (Shin et al., 2020), leading to an increased incidence or prevalence of aging-related diseases, such as tumours, cardiovascular and cerebrovascular diseases, diabetes, Alzheimer’s disease, and Parkinson’s disease. Stem cells are a type of undifferentiated cells with self-renewal capacity and multipotent differentiation potential. By committing themselves to tissue-specific cells through proliferation and differentiation, and subsequently replacing, repairing and regenerating degenerated tissues, stem cells play an important role in maintaining tissue homeostasis and preventing and treating aging-related diseases. Hence, tissue and organ aging is the outcome of stem cell senescence, that is, the loss of stem cell self-renewal, proliferation, and differentiation potentials caused by intrinsic and extrinsic insults.

DNA damage is known to increase with cellular senescence as demonstrated by an increase in DNA damage foci [phosphorylated histone H2AX (γH2AX)] in senescent cells. Furthermore, accumulated DNA damage has been demonstrated to be associated with cell senescence (Rossi et al., 2007; Hoeijmakers, 2009; Ou and Schumacher, 2018). Both cellular senescence (Ocampo et al., 2016) and DNA damage accumulation (Beerman et al., 2014) are considered the major causes of aging. Accordingly, the prevention, attenuation, or repair of DNA damage should be a significant strategy in attenuating stem cell cellular senescence. Although humans and other mammals have developed sophisticated and complex DNA repair systems, when DNA damage exceeds the ability of DNA repair, stem cells enter a state of aging, disrupt tissue homeostasis, and lead to age-related diseases. Therefore, the prevention or alleviation of DNA damage is an alternative to DNA repair in attenuating stem cell senescence and senescence-related diseases.

Pre B-cell leukaemia homeodomain (PBX) transcription factors belong to the PBC subgroup of three amino acid loop extension (TALE) homeodomain (HD)-containing proteins. PBX transcription factors are essential regulators of embryonic development, organogenesis, and foetal growth including morphogenesis, skeletal (Selleri et al., 2001; Capellini et al., 2006; Moens and Selleri, 2006), adipocyte, and neuron development (Jürgens et al., 2009; Grebbin et al., 2016; Villaescusa et al., 2016), cardiovascular differentiation (Chang et al., 2008; McCulley et al., 2017), and haematopoiesis (Ficara et al., 2008; Kocabas et al., 2015), contributing to concomitant patterning of the anterior-posterior body axis and conferring regional identity by regulating cell fate, in particular cell proliferation, apoptosis, senescence, and differentiation (Grebbin et al., 2016). PBX1 dimerizes with other TALE proteins via the highly conserved PBC domain to form nuclear complexes to enhance DNA protein binding and mark specific genes for activation by penetrating transcriptionally inactive chromatin regions and recruiting coactivators. Octamer-binding transcription factor 4 (OCT4), PBX1, and NANOG are well known core transcription factors that organize into a regulatory network that governs stem cell pluripotency and undifferentiated state (Chan et al., 2009). PBX1 transactivated NANOG promoter activity and synergistically interacted with OCT4 and SOX2 to maintain stem cells in an undifferentiated state.

The hair follicle is an appendage of human and mammalian skin (Wang et al., 2020a), derived from epithelial and mesenchymal cell interactions during embryogenesis (Ohyama and Veraitch, 2013; Tezuka et al., 2016; Ohyama, 2019). Hair follicle-derived stem cells coordinate in a temporal and spatial manner, leading to a hair cycle through which they maintain hair follicle homeostasis (Rompolas and Greco, 2014). Any damage to hair follicles may disrupt the hair follicle cycle, resulting in hair loss or hair greying, leading to depression, anxiety, or other mental health problems. As the outermost layer organs of the human body, hair follicles have a higher risk of exposure to the external environment compared to other organs and are thus, more vulnerable than are other organs to extrinsic insults. Accordingly, our recent study showed robust reactive oxygen species (ROS) accumulation and abrupt up-regulation of DNA damage and repair during hair cycle transition from the anagen to catagen phase (data not shown), suggesting a key role of ROS- mediated DNA damage and repair in hair cycle progression.

Hair follicle-derived mesenchymal stem cells (HF-MSCs) (Bai et al., 2017) participate in hair follicle genesis, repair, and regeneration, and hair cycle progression (Zhou et al., 2016). Moreover, because of the easily accessible and rich source of autologous stem cells (Li et al., 2020), HF-MSCs display remarkable advantages over other stem cell sources in stem cell-based regeneration medicine, in particular in hair regeneration (Wang et al., 2013). Any damages imposed on HF-MSCs may cause HF-MSC senescence, which not only disrupts hair follicle homeostasis, leading to hair loss, but also compromises the therapeutic potential of HF-MSCs, even raising safety concerns in HF-MSC-based regenerative medicine.

Our previous study showed that overexpression of PBX1 significantly attenuated HF-MSC senescence and apoptosis through activation of the downstream phosphatidylinositol 3-kinase (PI3K)/protein kinase B (AKT) (PI3K/AKT) signalling pathway (Jiang et al., 2019; Liu et al., 2019; Wang et al., 2020b). Whether PBX1 attenuates HF-MSC senescence through DNA damage and repair remains unclear. To this end we aimed to explore the effects of PBX1 on the cellular senescence of HF-MSCs insulted by intrinsic ROS or extrinsic H_2_O_2_. Our results showed that PBX1 overexpression attenuates HF-MSC senescence and apoptosis by alleviating reactive oxygen species (ROS)-mediated DNA damage instead of enhancing DNA repair. This is the first study to report that PBX1 attenuates stem cell senescence and apoptosis by alleviating DNA damage. It provides new insight into the mechanistic understanding of cellular senescence and apoptosis, and lays the foundation for developing relevant strategies for the alleviation of tissue and organ aging, in particular in hair regeneration.

## Materials and Methods

### Cell Culture

This study was approved by Ethnic Committee of School of Public Health, Jilin University (2021-06-06). The isolation and identification of HF-MSCs were performed in our previous study (Jiang et al., 2019; Liu et al., 2019; Shi et al., 2019). HF-MSCs were cultured in Dulbecco’s modified Eagle’s medium (Life Technologies, United States ) containing 10% fetal bovine serum (FBS, Hyclone, United States ), 2 ng/ml basic fibroblast growth factor (Sino Biological Inc., China) and 100 U/ml penicillin-streptomycin (Hyclone, Logan, UT, United States ). HEK293T cells were cultured in DMEM containing 10% FBS and 100 U/ml penicillin-streptomycin. The cells were cultured at 37°C and 5% CO_2_. When HF-MSCs or HEK293T proliferated to 80% confluence, they were digested with 0.25% trypsin and subcultured under the same individual conditions.

### Generation of the HF-MSCs Overexpressing PBX1, PARP1 and Both PBX1 and PARP1

The human PBX1 coding region and PARP1coding region was cloned into the pLVX-IRES-mCherry lentiviral vector (Youbio, China). The 10 μg lentiviral vector was cotransfected with 7.5 μg pMD2.G and 2.5 μg psPAX2 (Addgene) into 293T cells in a 100 mm cell culture plate using Lipofectamine (Invitrogen) 3,000 as transfection reagent. The viral particle were harvested at 48 and 72 h after transfection and concentrated by ultracentrifugation (Millipore, United States ). HF-MSCs were transduced with lentiviral particles encoding PBX1 or PARP1 or both PBX1 and PARP1 in the presence of polybrene (Santa Cruz, United States ) at final concentration of 10 μg/ml.

### Treatment HF-MSCs With H_2_O_2_

When HF-MSCs proliferate to 70–80% confluence in DMEM medium containing10% FBS, 2 ng/ml bFGF and 100 U/ml penicillin-streptomycin, they were incubated with indicated concentration of H_2_O_2_ for 2 h. After incubation, HF-MSCs were stained with Senescence-Associated-β-Galactosidase kit for cellular senescence assay or with apoptotic kit for apoptosis assay or released for Western blotting assay.

### Cell Proliferation and Cell Cycle Assays

HF-MSCs were plated at 1.8 × 10^4^ cells per well in a 24-well plate containing 90% DMEM and 10% FBS and cultured for 8 days to evaluate cell proliferation. Cells were washed with PBS, digested with 0.25% trypsin, and counted using a hemocytometer (Qiujing, Shanghai, China) at each day. For cell cycle assays, 1.2 × 10^6^ HF-MSCs were harvested and fixed in 70% ice-cold ethanol at -20°Covernight. Then HF-MSCs were washed three times with PBS and incubated in 500 μl of propidium iodide with RNase (BD Biosciences, United States ) for 15 min at room temperature in the dark. HF-MSCs were detect by flow cytometry (FACS Calibur flow cytometer; BD Biosciences, San Jose, CA, United States ) and analyzed by FlowJo software (Treestar, United States ). The cell proliferation index (PI) was calculated according to the following formula: PI = (S + G2/M)/(G0/G1 + S + G2/M) × 100*%.*

### Senescence-Associated-*β*-Galactosidase and Apoptosis Assays

For senescence-Associated-β-Galactosidase Assay, cellular senescence staining kit (Beyotime Biotechnology, China) was used to detect SA-*β*-gal activity positive cells according to instruction. Briefly when HF-MSCs reached 75–80% confluence in a 24-well plate, they were fixed for 15 min at room temperature and washed using PBS. Then the cells were incubated in Staining Solution Mix overnight at 37°C. Next day, cells were washed three times with PBS and observed using microscope (Leica, Germany) The number of β-Gal positive cells and total cells were counted from three fields of view randomly select.

For apoptosis assay, Annexin V-FITC/7-AAD Apoptosis Detection Kit (Sungene, China) was used to detect the apoptotic cells according to instruction Briefly 1 × 10^5^ HF-MSCs were suspended in 100 μl binding buffer containing 5 μl Annexin V-FITC and incubated for 15 min in the dark at room temperature. After incubation,5 μl 7-AAD was added and co-incubated for an additional 5 min at the same condition. Cells were then detected by flow cytometry.

### Immunofluorescence Staining and Flow Cytometry Assay

For immunofluorescence staining, HF-MSCs were fixed with 4% paraformaldehyde for 20 min at room temperature, blocked with 1% bovine serum albumin (Roche Diagnostics, France), and incubated with primary antibodies against PBX1 (Cell Signaling Technology, United States) at 4°C overnight. Next day Alexa Fluor 594-conjugated goat anti-rabbit antibodies (Cell Signaling Technology, United States) were used to detect the primary antibodies. HF-MSCs were then counterstained with DAPI (Life Technologies, United States) and imaged using fluorescence microscopy (Olympus, Japan). For ROS probing, HF-MSCs were collected by centrifugation, washed with PBS, and incubated with DCFH-DA (Beyotime Biotechnology, China). HF-MSCs were then detected by flow cytometry (BD, United States) and analyzed by FlowJo software (Treestar. United States).

### Western Blotting Assay

8 × 10^5^ HF-MSCs were plated in a 100 mm cell culture dish and culture in DMEM medium containing 10% FBS and 2 ng/ml bFGF. When HF-MSCs reached 80% confluence, they were harvested and lysed in 200 μl RIPA buffer (Beyotime Biotechnology, China) supplemented with 1% protease inhibitor cocktail (CoWin Biosciences, China) and 1% phosphatase inhibitor cocktail (CoWin Biosciences, China) at 4°C for 40 min, and centrifuged at 13,000 g for 20 min at 4°C. The supernatant was collected and protein concentration was analyzed using an Enhanced BCA Protein Assay Kit (Beyotime Biotechnology, China). 25 μg of protein per sample was loaded in each well and separated by 10% SDS polyacrylamide gel electrophoresis and then transferred to polyvinylidene difluoride membranes (Millipore, United States ). The membranes were incubated in 5% nonfat milk powder (Anchor, New Zealand) at room temperature for 45 min. The membranes were then incubated with primary antibodies:PBX1 rabbit mAb (1: 1000, CST), p16 rabbit mAb (1: 1000; ProteinTech Group Inc., United States), p21Waf1/Cip1 (12D1) rabbit mAb (1: 1000; CST), p53 mouse mAb (1: 1000; Santa Cruz Biotechnology, Inc.), PARP1 rabbit mAb (1: 1000; CST.), AIF rabbit mAb (1: 1000, CST), Sirtuin 1 mouse mAb (1: 1000, CST), Cleave Caspase 3 rabbit mAb (1: 1000, CST), γH2AX rabbit mAb (1: 1000, CST), GAPDH mouse mAb (1: 10000; ProteinTech Group Inc.), HRP conjugated AffiniPure Goat Anti-Rabbit IgG (H + L) (1: 5000; ProteinTech Group Inc.), and HRP-conjugated AffiniPure Goat Anti-Mouse IgG (H + L) (1: 5000; Protein-Tech Group Inc.). The proteins were visualized using a chemiluminescence imaging analysis system (ECL; Tanon 5200; Shanghai Tianneng Technology Co., Ltd., Shanghai, China), and band intensity was analyzed with Image J.

### Statistical Analysis

Data are statistically analyzed using SPSS software. Results are expressed as mean ± standard deviation and are representative of at least three independent experiments. Comparisons between the two groups were performed with independent sample t tests, and differences among multiple groups were compared with one-way analysis of variance. The results were considered significant at *p* < 0.05.

## Results

HF-MSC entry into replicative senescence and apoptosis is accompanied by increased ROS accumulation, DNA damage aggravation, and decreased PBX1 expression.

**FIGURE 1:**
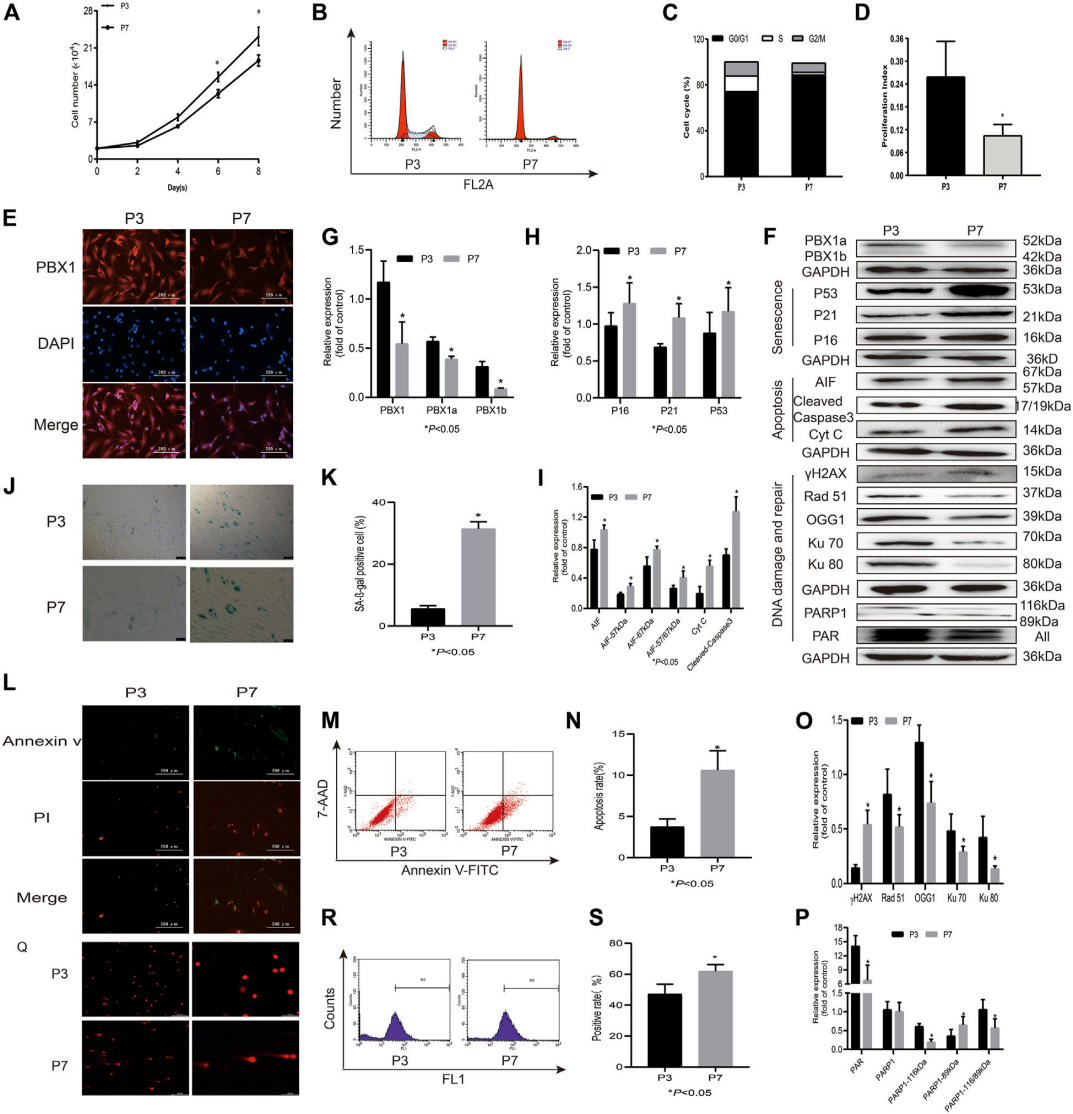
HF-MSCs enter replicative senescence and apoptosis accompanied by ROS accumulation, DNA damage aggravation, and reduced PBX1 expression. **(A)** Growth curve of HF-MSCs at P3 and P7. **(B)** Cell cycle of HF-MSCs in P3 and P7. **(C)** Cell cycle distribution of HF-MSCs at P3 and P7. **(D)** Proliferation index of HF-MSCs at P3 and P7. **(E)** Immunofluorescence analysis of PBX1 expression and localization in HF-MSCs (scale bar, 200 μm). **(F–I,O,P)** Western blotting results showing the protein expression levels of PBX1, Ku70, Ku80, Rad 51, γH2AX, PAR, and PARP1, P53, P21, P16, AIF, cleaved caspase 3, and Cyt C in HF-MSCs. **(J,K)** SA-β-gal staining results of HF-MSCs in P3 and P7. **(L)** Annexin V/PI staining results of HF-MSCs at P3 and P7 (scale bar, 200 μm). **(M,N)** Flow cytometry results of HF-MSC apoptosis in P3 and P7. **(Q)** Comet assay results of HF-MSCs at P3 and P7. **(R,S)** DCFH-DA results of ROS in P3 and P7. HF-MSCs, hair follicle-derived mesenchymal stem cell; ROS, reactive oxygen species; PBX1, pre-B-cell leukaemia homeobox 1; P, passage; SA-β-gal, senescence-associated-β-galactosidase; DCFH-DA, dichloro-dihydro-fluorescein diacetate, AIF, apoptosis‐inducing factor, γH2AX, phosphorylated histone H2AX; PARP1, poly (ADP-ribose) polymerase 1.

DNA damage has been considered the main culprit of cell senescence and apoptosis, and intrinsic ROS are the key endogenous factors that cause DNA damage (Canli et al., 2017). As expected, with cell passaging 1) HF-MSCs lost their proliferation potential and entered an apoptotic and senescent state, as shown in Figure 1. The cell numbers and cell proliferation index of HF-MSCs cultured in a 24-well plate significantly decreased from 2.3 × 10^5^ cells at P3 to 1.8 × 10^5^ at P7 (*p* < 0.05) on the 8th day of culture and from 25.90% at P3 to 10.49% at P7 (*p* < 0.05), respectively, (Figures 1A–D). The percentage of SA-β-gal-positive cells increased from 7.68% at P3 to 29.4% at P7 (*p* < 0.05; Figures 1J,K) and that of 7-AAD Annexin V-FITC-positive cells increased from 3.71% at P3 to 10.65% at P7 (*p* < 0.05; Figures 1L–N). In agreement with the results of the senescence and apoptosis assays, the western blotting assay showed that the expression levels of the senescence-associated proteins P16, P53, and P21 (*p* < 0.05; Figures 1F,H), and apoptosis-associated proteins Cyt C and cleaved caspase 3, were increased at P7 (*p* < 0.05; Figures 1F,I) compared to P3; 2) ROS accumulation and DNA damage aggravation were observed in HF-MSCs, as shown by the increased percentage of ROS-positive cells probed by DCFH-DA at P7 compared to P3 (*p* < 0.05; Figures 1R,S) and by the increased percentage of tailed DNA-positive cells (Figure 1Q). Western blot assay results showed that the expression of γH2AX and that of both 67 and 57 kDa AIF in HF-MSCs increased with cell passaging (*p* < 0.05; Figures 1F,I,O); 3), and the expression of DNA repair-related proteins Ku70, Ku80, Rad 51, PAR, and 116 kDa PARP1 in HF-MSCs decreased with cell passaging (*p* < 0.05; Figures 1F,O,P). In contrast, the expression of 89 kDa PARP1 in HF-MSCs significantly increased with cell passaging (*p* < 0.05; Figures 1F,P). In agreement with the results of immunofluorescence analysis, very promisingly, the western blotting assay results showed that PBX1 expression in HF-MSCs at P3 was significantly higher than that at P7 (*p* < 0.05; Figures 1E–G).

### PBX1 Overexpression Attenuates HF-MSC Senescence and Apoptosis Accompanied by Reduced ROS Accumulation and Down-Regulated DNA Damage and Repair-Related Protein Expression

**FIGURE 2:**
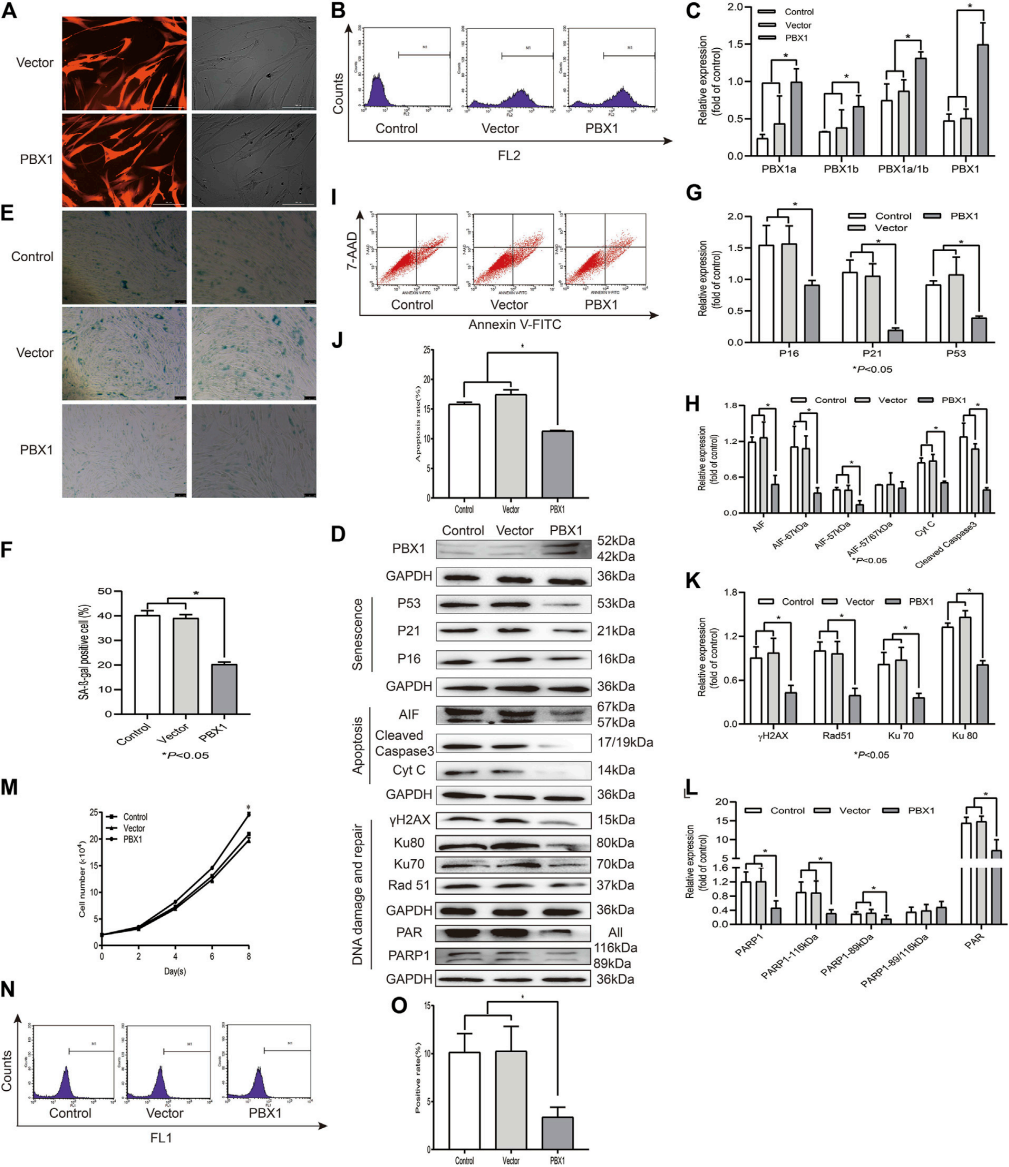
PBX1 attenuates cellular senescence and apoptosis in HF-MSCs accompanied by reduced ROS generation and down-regulated DNA damage and repair-related protein expression. **(A)** Images showing PBX1 overexpression after lentiviral transduction (scale bar, 200 μm). **(B)** Flow cytometry results of PBX1 expression in HF-MSCs after transduction. **(C)** Western blotting analysis of the protein expression level of PBX1 after lentiviral transduction. **(C,D,G,H,K,L)** Western blotting results of the protein expression levels of PBX1, Ku70, Ku80, Rad 51, γH2AX, PAR, and PARP1, P53, P21, P16, AIF, cleaved caspase 3, and Cyt C in HF-MSCs after PBX1 overexpression. **(E,F)** SA-β-gal staining results after PBX1 overexpression. **(I,J)** Flow cytometry results of HF-MSC apoptosis after PBX1 overexpression. **(M)** Growth curve in PBX1-overexpressing HF-MSCs. **(N,O)** Flow cytometry results of the ROS level in HF-MSCs after PBX1 overexpression. HF-MSCs, hair follicle-derived mesenchymal stem cell; ROS, reactive oxygen species; PBX1, pre-B-cell leukaemia homeobox 1; PARP1, poly [ADP-ribose] polymerase 1, AIF, apoptosis‐inducing factor.

Cellular senescence, apoptosis, and necrosis are the sequential consequences of cell death (Galluzzi et al., 2018). PBX1 is a pioneering transcription factor that participates in embryonic development (Capellini et al., 2006), organogenesis (Selleri et al., 2001; Moens and Selleri, 2006), maintenance of stem cell self-renewal (Ficara et al., 2008), and attenuation of stem cell senescence. As shown in Figure 2, our SA-*β*-gal staining and flow cytometry assay results showed that the percentages of SA-*β*-gal-positive cells or apoptotic cells in HF-MSCs overexpressing PBX1 were significantly lower than those of the empty vector group (*p* < 0.05; Figures 2E,F,I,J). Western blotting assay results showed that the expression of apoptosis-related proteins Cyt C, cleaved caspase 3, 57 and 67 kDa AIF and that of cellular senescence-associated proteins P53, P21, and P16 in HF-MSCs overexpressing PBX1 was significantly lower than that of the control or empty vector group (*p* < 0.05; Figures 2D,G,H).

As ROS activation, aggravated DNA damage, and reduced DNA repair are the major causes of cellular senescence and apoptosis (Canli et al., 2017), we aimed to find out whether PBX1-attenuated HF-MSC senescence and apoptosis is involved in ROS alleviation and DNA damage attenuation. As expected, PBX1 overexpression significantly reduced ROS generation and DNA damage, as shown by the reduced percentage of ROS-positive cells and of γH2AX expression. Surprisingly, compared with the empty vector and control groups (*p* < 0.05; Figures 2D,I,K), PBX1 overexpression down-regulated DNA repair-related protein expression (89 and 116 kDa PARP1, Ku 70, Ku 80, Rad51, and PAR) (*p* < 0.05; Figures 2D,K,L), suggesting that DNA damage reduction rather than DNA repair may be the major, if not unique, mechanism underlying the attenuation of cellular senescence and apoptosis in HF-MSCs by PBX1.

### Enhancement of Cellular Senescence and Apoptosis in HF-MSCs Induced by PARP1 Overexpression Is Correlated With Increased ROS Accumulation and Up-Regulated DNA Damage and Repair-Related Protein Expression

**FIGURE 3:**
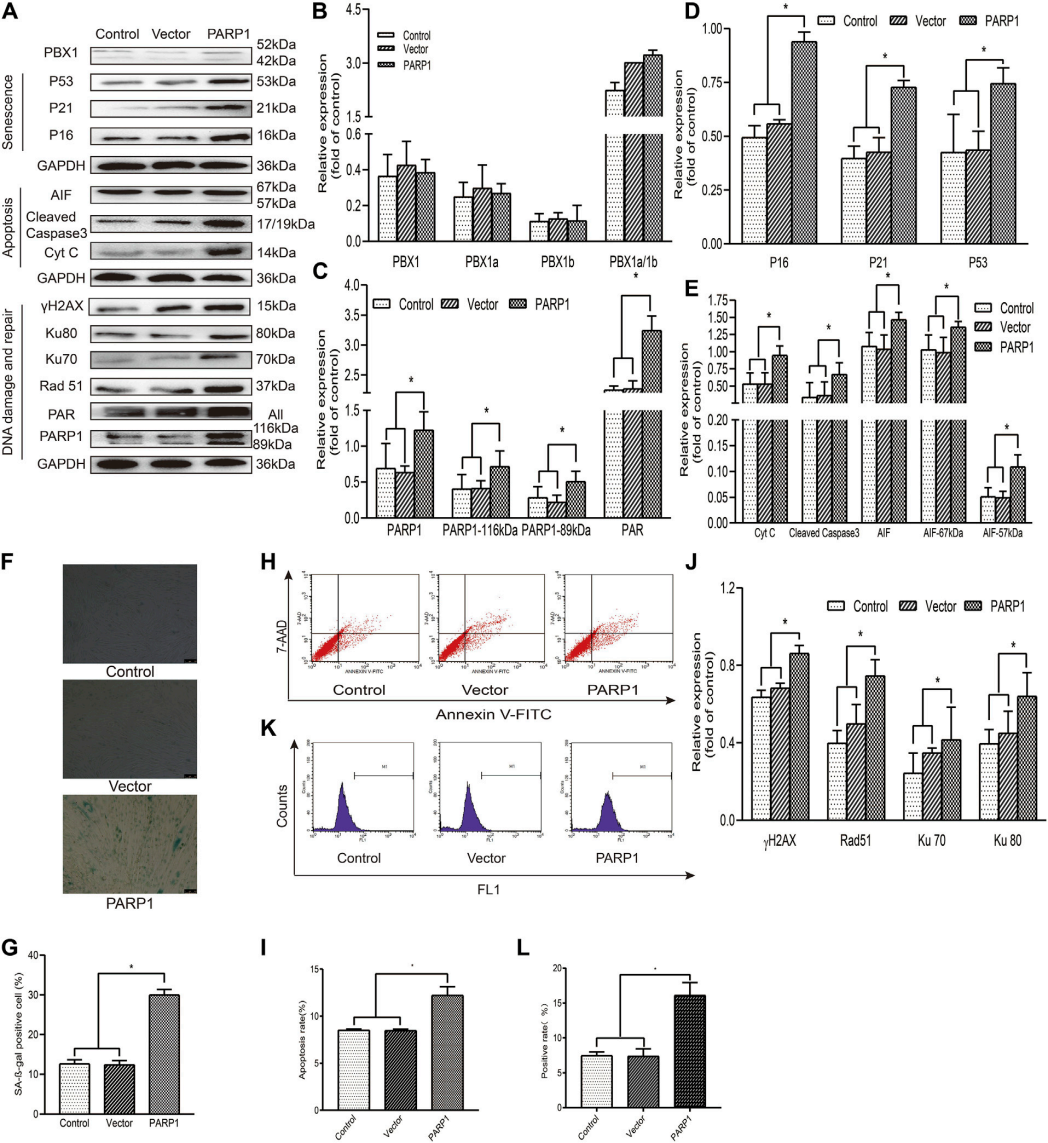
PARP1 aggravates senescence and apoptosis in HF-MSCs. **(A–E,J)** Western blotting analysis of the protein expression levels of PBX1, P53, P21, P16, AIF, cleaved caspase 3, Cyt C, Ku70, Ku80, Rad 51, γH2AX, PAR, and PARP1 after PARP1 overexpression. **(F,G)** HF-MSC SA-β-gal staining results after PARP1 overexpression. **(H,I)** Flow cytometry results of HF-MSC apoptosis after PARP1 overexpression. **(K,L)** Flow cytometry results of the ROS level in HF-MSCs after PARP1 overexpression. HF-MSCs, hair follicle-derived mesenchymal stem cell; ROS, reactive oxygen species; PBX1, pre-B-cell leukaemia homeobox 1; PARP1, poly (ADP-ribose polymerase 1, AIF, apoptosis‐inducing factor; SA-β-gal, senescence-associated-β-galactosidase.

PARP1 is the most abundant pleiotropic enzyme in the PARP family and participates in numerous critical cellular processes, such as DNA repair and AIF-mediated apoptosis (parthanatos) (Fatokun et al., 2014). To explore the role of PARP1 in HF-MSC senescence and apoptosis, we generated PARP1-overexpressing HF-MSCs. Our results showed that PARP1 overexpression increased the percentage of SA-*β*-gal-positive cells (*p* < 0.05; Figures 3F,G), apoptotic cells (*p* < 0.05; Figures 3H,I), and ROS-positive cells (*p* < 0.05; Figures 3K,L). Western blotting results showed that PARP1 overexpression upregulated the expression of 1) senescence-related proteins P53, P21, and P16 (*p* < 0.05; Figures 3A,D); 2) apoptosis-related proteins cleaved caspase 3, Cyt C, 57 kDa AIF, and 67 kDa AIF (*p* < 0.05; Figures 3A,E); 3) DNA damage and repair proteins γH2AX, PARP1, PAR, Ku 70, Ku 80, and Rad51 (*p* < 0.05; Figures 3A,C,J).

### PBX1 Attenuates HF-MSC Senescence and Apoptosis Through Alleviation of ROS-Mediated DNA Damage Instead of Promotion of DNA Repair

**FIGURE 4:**
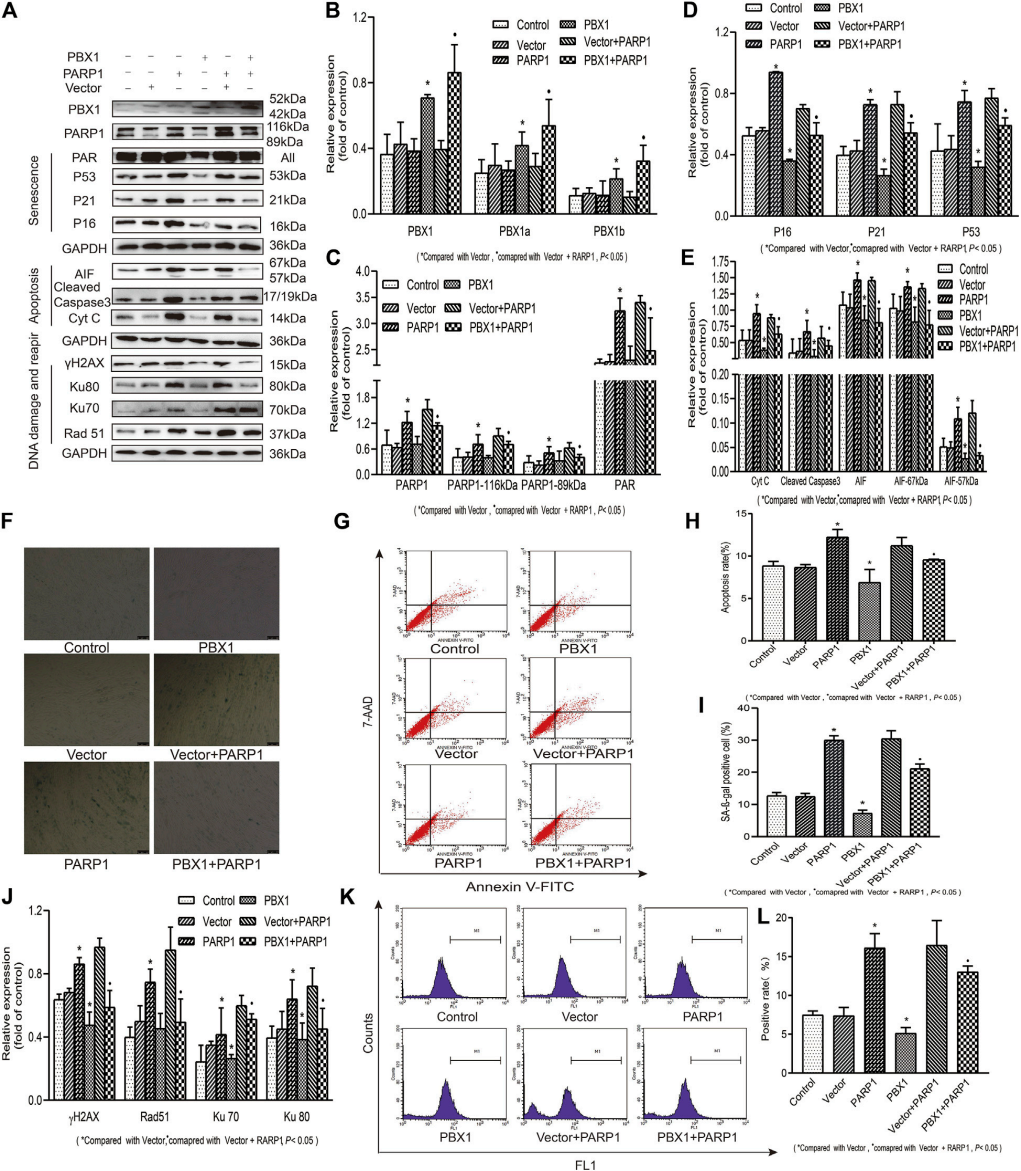
PBX1 attenuates cellular senescence and apoptosis of HF-MSCs by alleviating ROS-mediated DNA damage instead of enhancing DNA repair. **(A–E,J)** Western blotting analysis of the protein expression levels of PBX1, P53, P21, P16, AIF, cleaved caspase 3, Cyt C, Ku70, Ku80, Rad 51, γH2AX, PAR, and PARP1 after PBX1 overexpression in PARP1-overexpressing cells. **(F,I)** HF-MSC SA-β-gal staining results after PBX1 overexpression in PARP1-overexpressing cells. **(G,H)** Flow cytometry results of HF-MSC apoptosis after PBX1 overexpression in PARP1-overexpressing cells. **(K,L)** Flow cytometry results of the ROS level in HF-MSCs after PBX1 overexpression in PARP1-overexpressing cells. HF-MSCs, hair follicle-derived mesenchymal stem cell; ROS, reactive oxygen species; PBX1, pre-B-cell leukaemia homeobox 1; PARP1, poly (ADP-ribose) polymerase 1, AIF, apoptosis‐inducing factor; SA-β-gal, senescence-associated-β-galactosidase.

PARP1 has been shown to be a key regulator of DNA integrity maintenance. By relocating to DNA damage sites and recruiting DNA repair proteins, PARP1 participates in DNA repair through homologous recombinant repairing and non-recombinant end joining (Dulaney et al., 2017; Pascal, 2018). To clarify DNA damage alleviation or DNA repair, which affect the attenuation of cellular senescence and apoptosis of HF-MSCs, we generated PARP1-, PBX1-, and PARP1 + PBX1-overexpressing HF-MSCs (Figure 4) and compared the effects of PBX1-overexpression, PARP1-overexpression, and PARP1 + PBX1-overexpression on HF-MSC ROS activation, cellular senescence, and apoptosis. Our results showed that, compared with the empty vector group, PARP1 overexpression significantly increased the percentage of SA-*β*-gal-positive cells from 12.40 to 29.96% (*p* < 0.05; Figures 4F,I), that of apoptotic cells from 8.46 to 12.21% (*p* < 0.05; Figures 4G,H), and that of ROS-positive cells from 7.40 to 16.13% (*p* < 0.05; Figures 4K,L). In contrast, PBX1 overexpression decreased the percentages of SA-*β*-gal-positive cells (*p* < 0.05; Figures 4F,I), apoptotic cells (*p* < 0.05; Figures 4G,H), and ROS-positive cells (*p* < 0.05; Figures 4K,L) compared to those of the empty vector. Furthermore, both PARP1 and PBX1 overexpression decreased the percentages of SA-*β*-gal-positive cells (*p* < 0.05; Figures 4F,I), apoptotic cells (*p* < 0.05; Figures 4G,H), and ROS- positive cells (*p* < 0.05; Figures 4K,L), compared to those of vector + PARP1 overexpression. Western blot assay results showed that 1) PARP1 overexpression significantly up-regulated the expression of senescence-related proteins P53, P21, and P16 (*p* < 0.05; Figures 4A,D), apoptosis-related proteins 67 and 57 kDa AIF, cleaved caspase 3 and Cyt C (*p* < 0.05; Figures 4A,E), and DNA damage and repair-related proteins γH2AX, 116 kDa PARP1, PAR, Ku 70, Ku 80, and Rad51 compared to that of empty vector overexpression (*p* < 0.05; Figures 4A,C,J); 2) PBX1 overexpression significantly down-regulated the expression levels of senescence-related proteins P53, P21, and P16 (*p* < 0.05; Figures 4A,D), and of apoptosis-related proteins 57 kDa AIF, cleaved caspase 3 and Cyt C compared to those of empty vector overexpression (*p* < 0.05; Figures 4A,E); 3). Both PARP1 and PBX1 overexpression up-regulated PBX1 (*p* < 0.05; 4A, 4B), down-regulated the expression of senescence-related proteins: P53, P21, and P16 (*p* < 0.05; Figures 4A,D), apoptosis-related protein expression: 57 kDa AIF, cleaved caspase 3 and Cyt C (*p* < 0.05; Figures 4A,E), and DNA damage and repair-related proteins: γH2AX, PAR, Ku 70, Ku 80, and Rad 51 (*p* < 0.05; Figures 4A,C,J) compared to those of PARP1 vector overexpression, and the percentage of ROS positive cells was also decreased (*p* < 0.05; Figures 4K,L). These data suggested that PBX1 attenuated HF-MSC senescence and apoptosis by alleviating PARP1-mediated DNA damages instead of promoting DNA repair.

### PBX1 Attenuated H_2_O_2_-Induced Cellular Senescence and Apoptosis of HF-MSCs by Reducing ROS Accumulation and Alleviating DNA Damage

**FIGURE 5:**
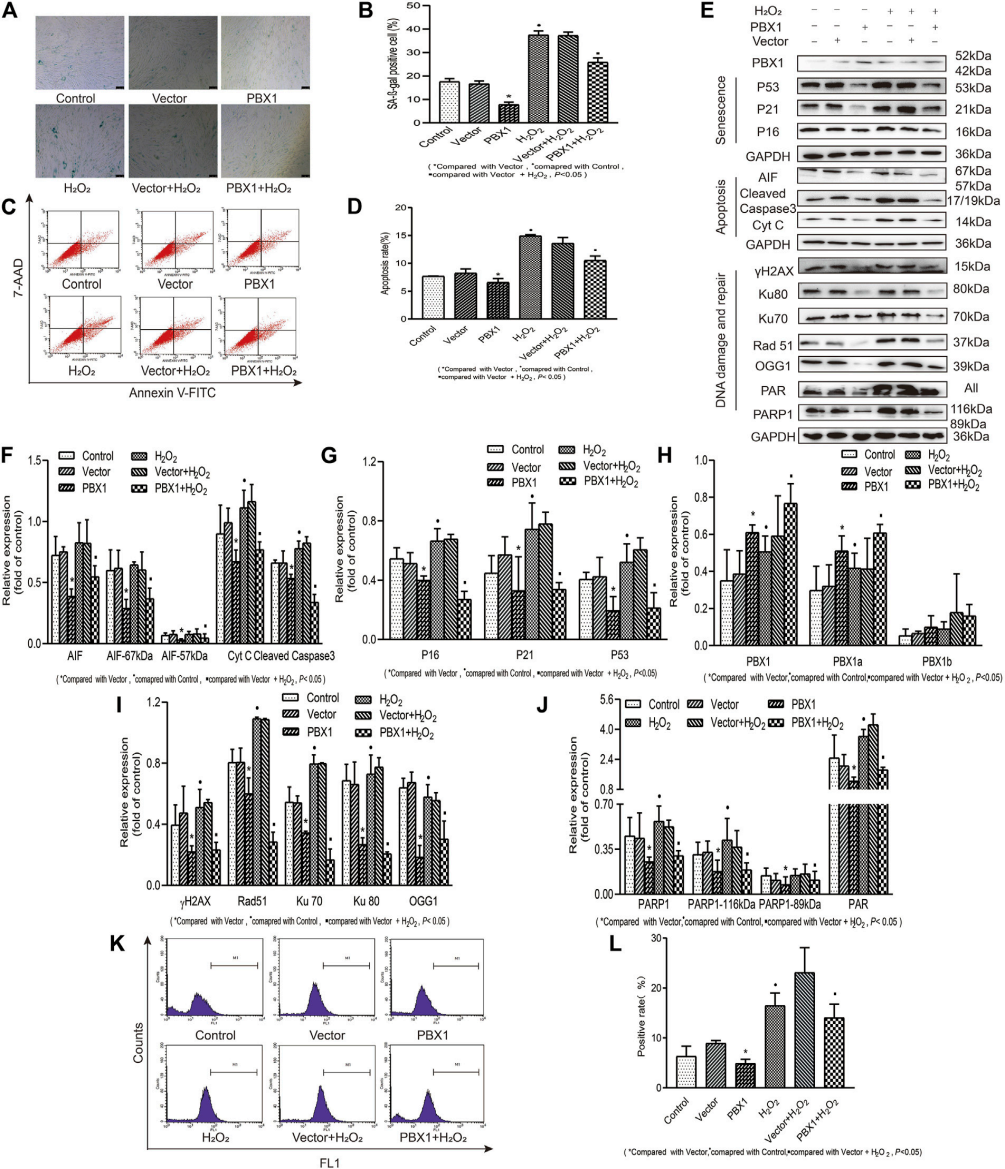
PBX1 attenuates H_2_O_2_-induced cellular senescence and apoptosis in HF-MSCs by alleviating ROS generation and DNA damage. **(A,B)** SA-β-gal staining results of H_2_O_2_-treated HF-MSCs after PBX1 overexpression. **(C,D)** Flow cytometry results of apoptosis in H_2_O_2_-treated HF-MSCs after PBX1 overexpression. **(E–J)** Western blotting analysis of the protein expression levels of PBX1, P53, P21, P16, AIF, cleaved caspase 3, Cyt C, Ku70, Ku80, Rad 51, γH2AX, PAR, and PARP1 in H_2_O_2_-treated HF-MSCs after PBX1 overexpression. **(K,L)** Flow cytometry results of the ROS levels in H_2_O_2_-treated HF-MSCs after PBX1 overexpression. HF-MSCs, hair follicle-derived mesenchymal stem cell; ROS, reactive oxygen species; PBX1, pre-B-cell leukaemia homeobox 1; PARP1, poly (ADP-ribose) polymerase 1, AIF, apoptosis‐inducing factor; SA-β-gal, senescence-associated-β-galactosidase.

In view of the fact that hair follicles are located on the surface of the human body, they are exposed to external environmental hazards, which may elicit oxidative damages in hair follicles and lead to hair follicle aging. Whether PBX1 attenuates HF-MSC extrinsic insult-induced cellular senescence and apoptosis and how HF-MSC senescence and apoptosis are attenuated remain unclear. To this end, we treated HF-MSCs with H_2_O_2_ to mimic the effects of extrinsic oxidative damage on HF-MSCs and explored the effects of H_2_O_2_ on HF-MSCs senescence and apoptosis and the related mechanism. Our results (Figure 5) showed that H_2_O_2_ treatment significantly increased the percentage of ROS-positive cells from 6.33 to 16.47% (*p* < 0.05; Figures 5K,L), that of apoptotic cells from 7.72 to 14.96% (*p* < 0.05; Figures 5C,D) and that of SA-*β*-gal staining gal-positive cells from 17.53 to 37.43% (*p* < 0.05; Figures 5A,B). In contrast, PBX1 overexpression in H_2_O_2_-treated and -untreated HF-MSCs significantly decreased the percentage of ROS-positive cells from 8.95 to 4.91% (*p* < 0.05; Figures 5K,L), and from 23.10 to 14.07% (*p* < 0.05; Figures 5K,L), that of apoptotic positive cells from 8.21 to 6.03% (*p* < 0.05; Figures 5C,D), and from 14.06 to 10.04% (*p* < 0.05; Figures 5C,D), and that of SA-*β*-gal-positive cells from 16.61 to 17.76% (*p* < 0.05; Figures 5A,B) and from 37.16 to 25.80% (*p* < 0.05; Figures 5A,B). In agreement with ROS, senescence, and apoptosis assay results, the western blotting assay showed that PBX1 overexpression in H_2_O_2_-treated and -untreated HF-MSCs significantly reduced the expression of 1) senescence-related proteins P53, P21, and P16 (*p* < 0.05; Figures 5E,G); 2) DNA damage and repair-related proteins γH2AX, OGG1, Rad51, KU70, Ku80, PAR, and 116 kDa PARP1 (*p* < 0.05; Figures 5E,I,J); and 3) apoptosis related proteins 57 and 67 kDa AIF, cleaved caspase 3, and Cyt C (*p* < 0.05; Figures 5E,F). These data suggest that PBX1 attenuated HF-MSC H_2_O_2_-induced cellular senescence and apoptosis by alleviating ROS generation and DNA damage instead of by promoting DNA repair.

## Discussion

In this study, we demonstrate that PBX1 overexpression enhances HF-MSC proliferation and attenuates cellular senescence and apoptosis by alleviating ROS-mediated DNA damage instead of promoting DNA repair. Cellular senescence includes replicative senescence and stress-induced premature senescence. DNA damage is a common cause of both senescence types. DNA damage activates cyclin-dependent kinase inhibitor P16 and P53 (Tchkonia et al., 2013; Muñoz-Espín and Serrano, 2014; Barnes et al., 2019) and the downstream signalling pathway, leading to cell-cycle arrest, cellular apoptosis, and senescence (Stewart and Weinberg, 2006; Shimizu et al., 2014). Furthermore, P16 and P53 interact with each other and subsequently activate cyclin-dependent kinase inhibitor p21, leading to cell cycle arrest through inhibition of cyclin-dependent kinase-2/4. The p53/p21 and p16/Rb signalling pathways are known to be involved in the regulation of cellular senescence, and p53 protein, the best-characterized transcriptional factor mediating DNA damage response, contributes to maintaining genomic stability and integrity through inducing apoptosis and inhibiting tumorigenesis (Shimizu et al., 2014). In this study, subculturing or treating HF-MSCs with H_2_O_2_ increased the percentage of β-gal-positive cells and apoptotic cells, suggesting that, after subculturing or H_2_O_2_ treatment, HF-MSCs enter cellular senescence and apoptosis, accompanied by ROS accumulation and increased expression of proteins related to cellular senescence, apoptosis, and DNA damage and repair.

PARP1 is a well characterized protein accounting for the majority of PARylation (Kadam et al., 2020). By catalysing the transfer of the ADP-ribose moiety from NAD to target proteins, PARP1 exhibits diverse cellular functions depending on the nature and strength of extrinsic and intrinsic stress stimuli. PARP1 exhibits dual functions; on the one hand it maintains DNA integrity by relocating to the DNA damage site and recruiting DNA repair molecules to initiate DNA repair, and on the other hand, over-activated PARP1 induces cell death by inducing AIF nucleus translation, large DNA fragmentation and ATP completion. PBX1 is a pioneering transcription factor participating in embryonic development, organogenesis, and foetal growth. By cooperating with Oct 4, Nanog, and Sox2, PBX1 induces the expression of multiple target genes related to pluripotency and multipotency, maintains stem cell self-renewal and proliferation potential. Our recent studies showed that by interacting with Nanog, PBX1 enhances HF-MSC proliferation and reprograming into induced pluripotent stem cells and attenuates HF-MSC senescence (Jiang et al., 2019). Here, we found that PBX1 expression reduced with HF-MSC passaging, suggesting that PBX1 may be involved in HF-MSC senescence. To explore whether PBX1 participates in HF-MSC attenuation of cellular senescence and apoptosis and to reveal the related underlying mechanism, we generated HF-MSCs over-expressing PBX1, PARP1, or both. As expected, PBX1 overexpression significantly reduced ROS accumulation, cellular senescence, and apoptosis in HF-MSCs, suggesting that PBX1 participates in the attenuation of cellular senescence and apoptosis in HF-MSCs possibly through interfering with ROS-mediated DNA damage and repair. In contrast, PARP1 overexpression increased ROS accumulation and cellular senescence and apoptosis in HF-MSCs, which correlated with the increased expression of proteins related to cellular senescence (P53, P16, and P21), apoptosis (57 kDa AIF, cleaved caspase 3 and Cyt C) and DNA damage and repair (γH2AX, Rad 51, Ku70, Ku 80, PAR, and 116 kDa PARP1). However, PARP1 overexpression did not change PBX1 expression, suggesting that PBX1 may be upstream of PARP1. Interestingly, compared with PARP1 + vector overexpression, both PBX1 and PARP1 overexpression reduced ROS accumulation and cellular senescence and apoptosis in HF-MSCs, consistent with the western blotting results. Both PBX1 and PARP1 overexpression up-regulated PBX1 expression, down-regulated proteins related to cellular senescence, apoptosis, and DNA damage and repair. In view of the fact that PBX1 overexpression down-regulated PRPA1 expression, and PARP1 overexpression did not change PBX1 expression, PBX1 may be upstream of PARP1. In this study, PARP1 overexpression in HF-MSCs exhibited similar cellular functions to those of PARP1 + Vector overexpression, suggesting that vector transduction did not change cellular functions. As hair follicles are located on the outmost layer of the skin, they are exposed to extrinsic insults. We treated HF-MSCs with H_2_O_2_ to mimic the effects of extrinsic insult-induced damages. Similar to intrinsic ROS effects, H_2_O_2_ treatment induced cellular senescence and apoptosis in HF-MSCs, which was accompanied by ROS accumulation and upregulated expression of proteins related to cellular senescence (P53, P16, and P21), apoptosis (57 kDa AIF, cleaved caspase 3, and Cyt C), and DNA damage and repair (γH2AX, Rad 51, Ku70, Ku80, PAR, and 116 kDa PARP1). In contrast, PBX1 overexpression reduced cellular senescence and apoptosis in H_2_O_2_-treated or -untreated HF-MSCs, which was accompanied by decreased ROS levels and down-regulated expression of proteins related to cellular senescence (P21, P16, and P53), apoptosis (57 and 67 kDa AIF, Cyt C and cleaved caspase 3), and DNA damage and repair (γH2AX, Rad 51, Ku70, Ku80, PAR and 89 and 116 kDa PARP1). Overall, our results showed that PBX1 overexpression attenuated cellular senescence and apoptosis in HF-MSCs by alleviating ROS-mediated DNA damage and repair, providing new insight into the mechanistic understanding of cellular senescence and apoptosis, and significantly contributing to the future development of strategies for alleviating tissue and organ aging, and, in particular, hair regeneration.

HF-MSCs are one of the major components of hair follicles. By interacting with other cell types in hair follicles in a temporal and spatial manner, HF-MSCs participate in hair genesis, repair, and regeneration, and in follicle cycle progression. Hair follicle homeostasis is maintained through the hair cycle. Any insults resulting in hair senescence or hair cycle disruption may lead to hair loss. Given that mammalian cells have limited DNA repair capacity, the development of a novel strategy to attenuate mammalian cell DNA damages attracts great attention in stem cell senescence research. Moreover, as hair follicles are an easily accessible and rich source of autologous stem cells, hair follicle-derived stem cells have more advantages than other stem cell sources in stem cell-based regenerative medicine (Jiang et al., 2019). Thus, the attenuation of HF-MSC senescence is of paramount significance in hair follicle regeneration and stem cell-based regenerative medicine.

**FIGURE 6:**
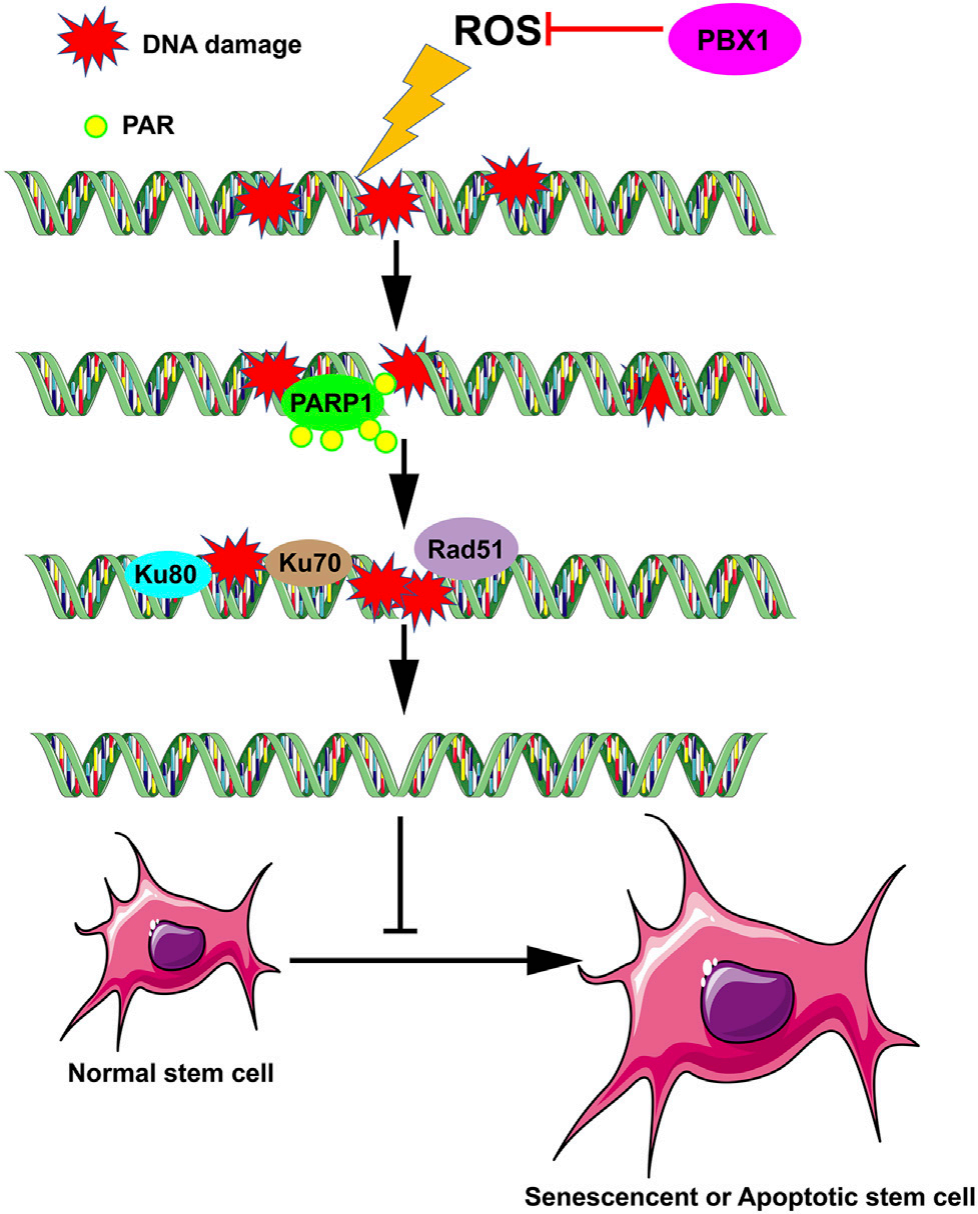
PBX1 attenuates hair follicle-derived mesenchymal stem cell senescence and apoptosis by alleviating ROS-mediated DNA damage instead of by enhancing DNA damage repair. ROS, reactive oxygen species; PBX1, pre-B-cell leukaemia homeobox 1.

In this study we demonstrate that PBX1 reduced cellular senescence and apoptosis in HF-MSCs by alleviating ROS-mediated DNA damage rather than promoting DNA repair, suggesting that the attenuation of stem cell senescence and apoptosis can be achieved by alleviating DNA damage (Figure 6). This study provides novel insight into the role of transcription factors in the attenuation of stem cell senescence, in particular the role of PBX1 in alleviating cellular senescence by alleviating DNA damages, and the relevant underlying mechanism. Moreover, it lays the foundation for developing relevant strategies for age-related disease prevention and treatment, and in particular, hair follicle repair and regeneration.

## Conclusion

In this study we demonstrated that PBX1 reduced senescence and apoptosis in HF-MSCs through the reduction of ROS-mediated DNA damage, rather than through DNA repair, suggesting that in stem cells, senescence and apoptosis can be attenuated by reducing DNA damage (Figure 6). This work provides support for a new mechanism of stem cell senescence and significantly contributes to efforts on age-related disease prevention and treatment, specifically, hair follicle repair and regeneration.

## Data Availability

The raw data supporting the conclusions of this article will be made available by the authors, without undue reservation.
